# Gene Expression Profiling Identifies HOXB4 as a Direct Downstream Target of GATA-2 in Human CD34+ Hematopoietic Cells

**DOI:** 10.1371/journal.pone.0040959

**Published:** 2012-09-24

**Authors:** Tohru Fujiwara, Hisayuki Yokoyama, Yoko Okitsu, Mayumi Kamata, Noriko Fukuhara, Yasushi Onishi, Shinichi Fujimaki, Shinichiro Takahashi, Kenichi Ishizawa, Emery H. Bresnick, Hideo Harigae

**Affiliations:** 1 Molecular Hematology/Oncology, Tohoku University Graduate School of Medicine, Sendai, Japan; 2 Hematology and Rheumatology, Tohoku University Graduate School of Medicine, Sendai, Japan; 3 Department of Hematology, Sendai Medical Center, Sendai, Japan; 4 Infection Control and Laboratory Diagnosis, Tohoku University Graduate School of Medicine, Sendai, Japan; 5 Division of Hematology, Kitasato University School of Allied Health Sciences, Sagamihara, Japan; 6 Department of Cell and Regenerative Biology, Wisconsin Institutes for Medical Research, University of Wisconsin School of Medicine and Public Health, Madison, Wisconsin, United States of America; Southern Illinois University School of Medicine, United States of America

## Abstract

Aplastic anemia is characterized by a reduced hematopoietic stem cell number. Although *GATA-2* expression was reported to be decreased in CD34-positive cells in aplastic anemia, many questions remain regarding the intrinsic characteristics of hematopoietic stem cells in this disease. In this study, we identified *HOXB4* as a downstream target of GATA-2 based on expression profiling with human cord blood-derived CD34-positive cells infected with control or *GATA-2* lentiviral shRNA. To confirm the functional link between *GATA-2* and *HOXB4*, we conducted GATA-2 gain-of-function and loss-of-function experiments, and *HOXB4* promoter analysis, including luciferase assay, in vitro DNA binding analysis and quantitative ChIP analysis, using K562 and CD34-positive cells. The analyses suggested that GATA-2 directly regulates *HOXB4* expression through the GATA sequence in the promoter region. Furthermore, we assessed *GATA-2* and *HOXB4* expression in CD34-positive cells from patients with aplastic anemia (n = 10) and idiopathic thrombocytopenic purpura (n = 13), and demonstrated that the expression levels of *HOXB4* and *GATA-2* were correlated in these populations (r = 0.6573, p<0.01). Our results suggested that GATA-2 directly regulates *HOXB4* expression in hematopoietic stem cells, which may play an important role in the development and/or progression of aplastic anemia.

## Introduction

The pathogenesis of aplastic anemia (AA) is complex and many questions remain unanswered. It has been proposed that immunological injury in hematopoietic stem cells (HSCs) leads to reduced numbers of stem cells in the bone marrow (BM) [1], [2], [3]. Evidence supporting this suggestion was obtained from in vitro analysis of samples obtained from AA patients, as well as from clinical evidence that immunosuppressive therapy with drugs such as anti-thymocyte globulin (ATG) and cyclosporin A is effective in 75% of AA cases [2], [3]. On the other hand, the observation that some patients do not respond to immunosuppressive therapy, and may even develop myelodysplastic syndrome or acute leukemia as a consequence of clonal evolution [4], suggests that the immunological aberration is not the sole mechanism of the disease. However, only limited information has emerged regarding potential intrinsic abnormalities of HSCs in AA, as most studies have focused on the extrinsic immunological abnormalities.

Hematopoiesis is intricately controlled by numerous transcription factors that control and coordinate the expression of lineage-specific genes [5]. Foundational studies defined involvement of members of a family of developmental regulators, the GATA transcription factors [6], [7], [8], [9]. GATA-1, GATA-2, and GATA-3 are termed as hematopoietic GATA factors, based on their important activities in controlling distinct and overlapping aspects of hematopoiesis [8]. GATA-1 functions to promote the development of erythrocytes, megakaryocytes, eosinophils, and mast cells, and GATA-3 plays a role in T-lymphocyte development [5], [8], [9], [10], [11]. On the other hand, GATA-2 functions early in hematopoiesis and is required for maintenance and expansion of HSCs and/or multipotent progenitors [9], [12], [13], [14], [15]. Therefore, it is possible that changes in the *GATA-2* expression level could lead to aberrant proliferation and differentiation of HSCs, and may be responsible for the development of AA. Previously, we demonstrated decreased expression of *GATA-2* in CD34-positive cells in AA [16]. This observation was confirmed in another study, which involved microarray-based transcriptional profiling in CD34-positive cells with AA [17]. Therefore, we speculated that some stem cell-specific genes are aberrantly expressed as a consequence of *GATA-2* downregulation in HSCs in AA, which could contribute to the development and/or progression of the disease.

**Figure 1:**
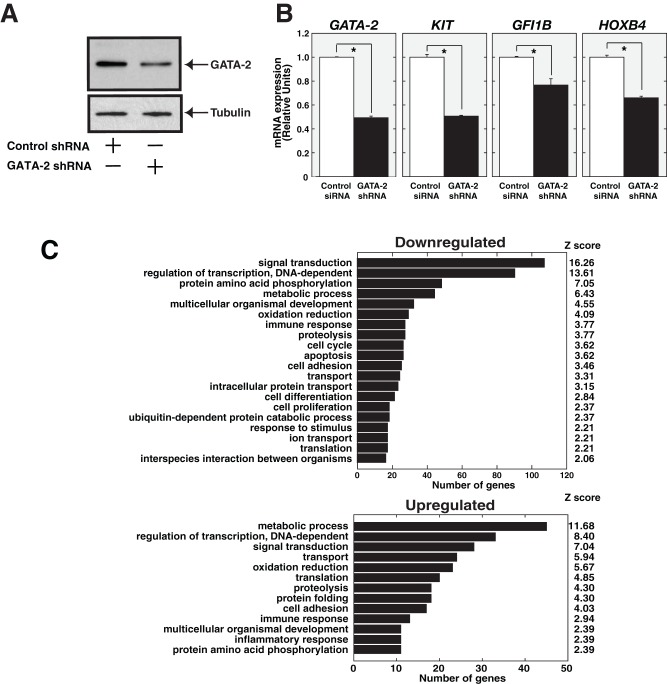
Expression profiling of GATA-2-regulated genes in CD34-positive cells. (A) GATA-2 knockdown in human cord blood-derived CD34-positive cells. Anti-GATA-2 Western blotting analysis of whole-cell extract from cord blood-derived CD34-positive cells, infected with control or GATA-2 shRNA, respectively.Alpha-Tubulin was used as a loading control. (B) Quantitative RT-PCR validation of array results (mean ± SE, n = 3). *28S* mRNA was quantified as a control. (C) Gene Ontology analysis. Genes showing greater than or equal to 1.4-fold change based on microarray analysis and greater than or equal to 20 normalized expression values were included in the analysis. A Z-value of 2.0 was used as the standard cut-off value.

To examine this possibility, we conducted microarray analysis with human cord blood-derived CD34-positive cells infected with lentiviral shRNA specific to *GATA-2*. Among GATA-2-regulated genes, we focused on the *HOXB4* gene as one of the causative candidate genes for AA. In the present study, we investigated the functional link between *GATA-2* and *HOXB4*, and also examined correlation between *GATA-2* and *HOXB4* expression based on clinical specimens.

**Figure 2:**
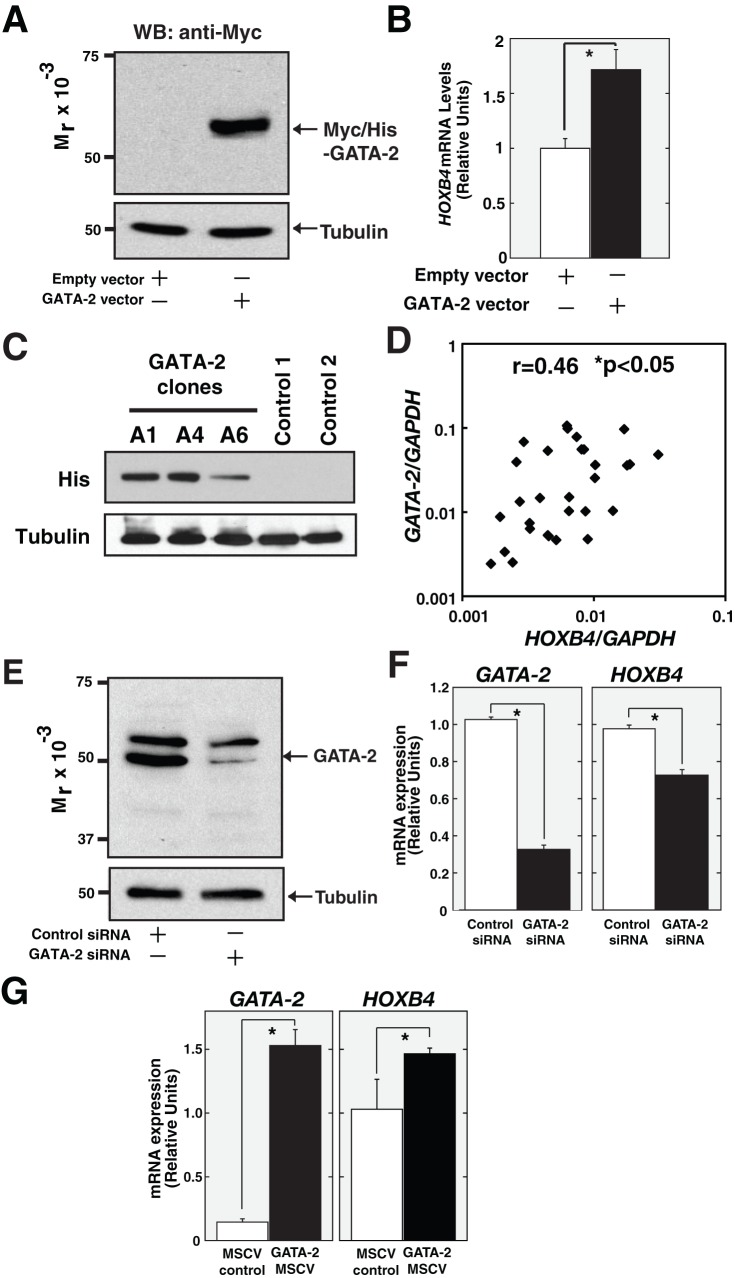
GATA-2 regulates *HOXB4* expression in K562 cells and CD34-positive cells. (A, B) Transient overexpression of GATA-2 in K562 cells. (A) Expression of Myc/His-tagged GATA-2 was confirmed by Western blotting. As a control, empty vector was independently transfected into K562 cells. Alpha-Tubulin was used as a loading control. (B) Quantitative RT-PCR analysis of *HOXB4* mRNA in K562 cells overexpressing GATA-2 (mean ± SD, n = 4). mRNA levels were normalized relative to *GAPDH*. *p<0.05. (C, D) Stable GATA-2 overexpression in K562 cells. GATA-2 expression vector was transduced into K562 cells and 28 clones were established after selection with G418 (Sigma). (C) Western blotting to detect stable expression of exogenous Myc/His-tagged GATA-2 in K562 cells. The results of 3 representative clones (A1, A4, and A6) and 2 control clones are shown. Alpha-Tubulin was used as a loading control. (D) Correlation between *GATA-2* and *HOXB4* mRNA in each clone. mRNA expression was normalized relative to that of *GAPDH*, and the correlation of these genes was assessed by Spearman's rank correlation method. (E–F) GATA-2 knockdown in K562 cells. (E) Anti-GATA-2 Western blotting analysis of whole-cell extracts and (F) Quantitative RT-PCR analysis of *GATA-2* and *HOXB4* (mean ± SE, n = 3), from K562 cells transfected with siRNA against human GATA-2 or control siRNA. Alpha-Tubulin was used as a loading control. mRNA levels were normalized relative to GAPDH. *p<0.05. (G) MSCV retroviral vector-mediated GATA-2 overexpression in cord blood-derived CD34-positive cells. Quantitative RT-PCR analysis was performed to detect *GATA-2* and *HOXB4* expression (mean ± SE, n = 3). mRNA levels were normalized relative to *28S*. *p<0.05.

## Materials and Methods

### Clinical samples

Patients involved in the analysis were diagnosed with AA (n = 10) or idiopathic thrombocytopenic purpura (ITP) (n = 13) in our institute or related hospitals [16]. The severity of AA at the time of diagnosis was moderate (n = 7) or severe (n = 3). Diagnosis and assessment of disease severity were performed according to published criteria [18]. Clinical samples were collected after obtaining written informed consent. The use of clinical samples as well as cord blood samples for the study was approved by the ethical committee of Tohoku University. Ethical considerations according to the declaration of Helsinki were followed.

**Figure 3:**
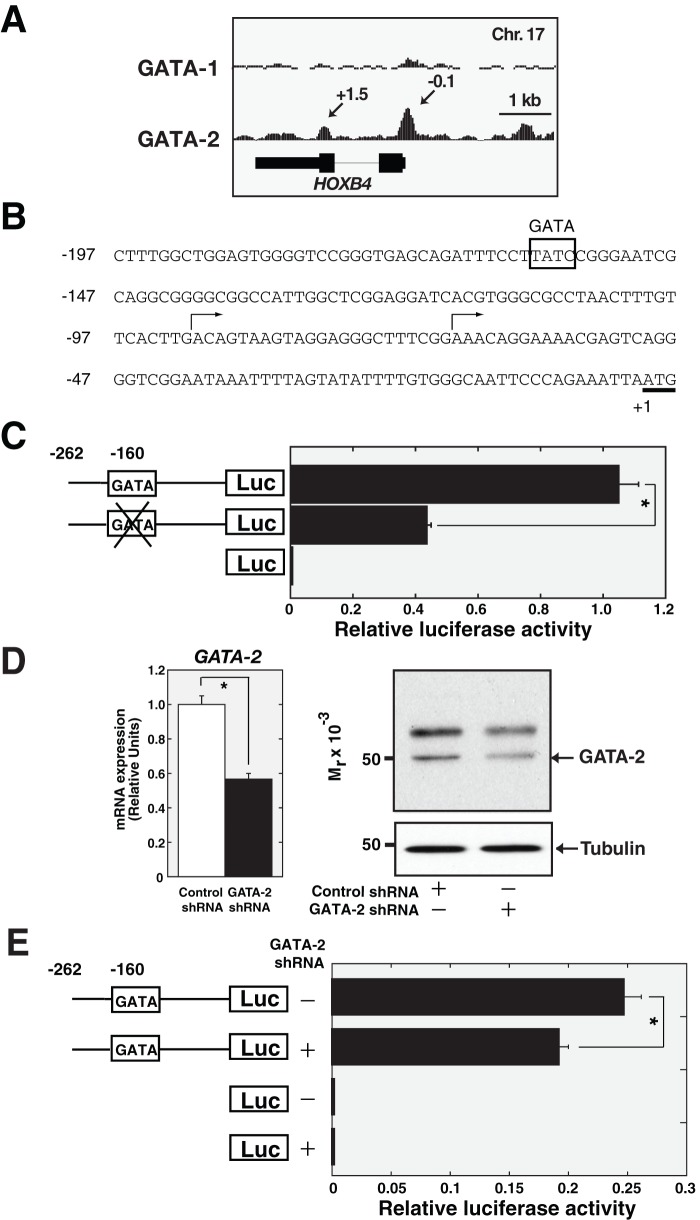
GATA-2 regulates *HOXB4* promoter activity. (A) GATA-1 and GATA-2 ChIP-seq profile at *HOXB4* in K562 cells. GATA-1 and GATA-2 signal map for HOXB4 was shown. Arrows, ChIP-seq peak locations relative to the start site of the respective GATA-1 target gene (kb). The ChIP-seq data were mined from data described by Fujiwara *et al* [24] and were analyzed with the integrated genome browser (IGB) (Affymetrix). (B) The 5′ upstream region of the human *HOXB4* gene. The putative GATA-binding element is boxed. The translational initiation site is underlined. Two transcriptional start sites [20] are indicated by arrows. (C) Impact of GATA site deletion on *HOXB4* promoter activity. We generated wild-type and −160/−157 GATA-deleted constructs fused to a luciferase reporter gene, and transient transfection assay was performed in K562 cells (mean ± SD, n = 3). *p<0.05. (D) GATA-2 knockdown in K562 cells. Anti-GATA-2 Western blotting analysis of whole-cell extract (left) and quantitative RT-PCR analysis of *GATA-2* (right) from K562 cells, infected with control or GATA-2 shRNA, respectively. Alpha-Tubulin was used as a loading control, and *GAPDH* mRNA was quantified as a control. (E) Impact of GATA-2 deletion on *HOXB4* promoter activity. HOXB4 promoter assay was conducted with K562 cells expressing control or GATA-2 shRNA (mean ± SD, n = 3). *p<0.05.

### Cell lines and isolation of CD34-positive cells

K562 cells were maintained in RPMI medium containing 10% fetal bovine serum. Methods for establishment of K562 cell lines stably expressing exogenous *GATA-2* were described previously [19]. GATA-2 expression vector was constructed using the coding region of human *GATA-2* cloned into pcDNA(−)/Myc-His vector (Invitrogen) [19].

**Figure 4:**
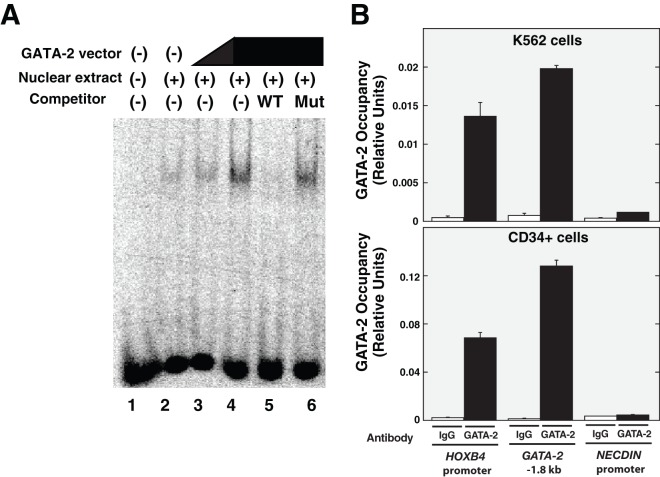
GATA-2 binds to *HOXB4* promoter. (A) Electrophoretic mobility shift assays. Aliquots of 3 ug of nuclear extracts from K562 (lane 2) or GATA-2 expression vector-transfected (lanes 3–6) K562 cells were incubated with FITC-labeled oligomers including the GATA site at −160/−157 within the HOXB4 promoter in the absence (lanes 1–4) or presence (lanes 5–6) of a 100-fold molar excess of the indicated oligonucleotides. For GATA-2 overexpression, pcDNA(−)/Mys-His expression vector was used (Figures 2A, 2B). (B) Quantitative ChIP analysis of GATA-2 chromatin occupancy at *HOXB4* promoter, *GATA-2* −1.8 kb, and *NECDIN* promoter, with K562 cells (upper) and cord blood-derived CD34-positive cells (lower) (mean ± SD, n = 3).

CD34-positive cells were isolated from BM cells obtained from patients and human cord blood-derived mononuclear cells using a magnetic activated cell sorter (MACS) system (Miltenyi Biotec). StemPro-34 SFM (Invitrogen) supplemented with Stem Cell Factor (100 ng/mL), IL-3 (50 ng/mL), and GM-CSF (25 ng/mL) (R&D systems) was used for expansion of isolated CD34-positive cells.

### Viral vectors and cell transduction

For *GATA-2* knockdown in CD34-positive cells and K562 cells, pGIPZ lentiviral shRNAmir (Open Biosystems) was used according to the manufacturer's protocol. After infection with CD34-positive cells, Puromycin (Sigma) was added to culture media for selection of the transduced cells for 96 h. For *GATA-2* overexpression, retroviruses encoding human *GATA-2*, and their MSCV control, were produced by transfection of Platinum Retroviral Packaging Cell Lines (Cell Biolabs) with FuGene HD (Promega). Seventy-two hours after transfection, the viral supernatant was used for infection. After infection with CD34-positive cells, cell were incubated for 48 h and then harvested for the analysis.

**Figure 5:**
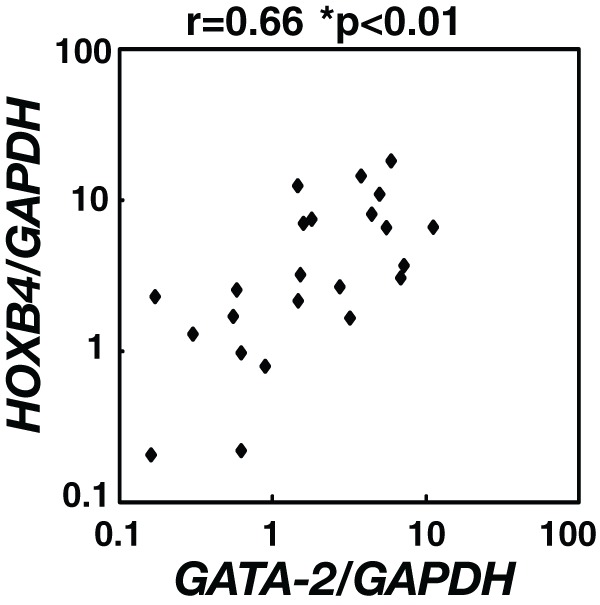
Significant correlation between *GATA-2* and *HOXB4* expression in CD34-positive cells in patients with AA and ITP. Bone marrow cells were collected from 10 patients with AA and 13 patients with ITP after obtaining informed consent [16]. Mononuclear cells were isolated by Ficoll-Hypaque density gradient centrifugation. CD34-positive cells were then collected using the MACS system. GATA-2 and HOXB4 mRNA levels were examined by quantitative RT-PCR analysis, and were normalized relative to *GAPDH* mRNA expression. The correlation of *HOXB4* and *GATA-2* mRNA expression in each sample was calculated with Spearman's rank correlation test.

### siRNA-mediated knockdown of GATA-2

The antisense sequence of the siRNA for human *GATA-2* was gugcugauguagugaccaaTT (B-Bridge International Inc.). AllStars Negative Control siRNA (Qiagen) was used as a control. A total of 200 pmol of siRNA was transfected into CD34-positive cells and K562 cells using an Amaxa Nucleofector kit (Amaxa Inc.). Cells were harvested after 24 h for CD34-positive cells and after 48 h for K562 cells (transfected twice at 0 and 24 h).

### Real-time quantitative PCR and expression profiling

Total RNA was purified with TRIzol (Invitrogen), and cDNA was synthesized from 1.5 ug of purified total RNA using Superscript II (Invitrogen). Reaction mixtures (20 uL) for real-time quantitative RT-PCR consisted of 2 uL of cDNA, 10 uL of Power SYBR green master mix (Applied Biosystems) and appropriate primers. Primer sequences are available upon request. Product accumulation was monitored by measuring SYBR Green fluorescence and normalized relative to *GAPDH* or *28S* mRNA.

For expression profiling, Human Oligo chip 25k (Toray) was used according to the manufacturer's protocol. Aliquots of 1 ug of total RNA were amplified with an Ambion Amino Allyl aRNA kit (Ambion), labeled with Amersham Cy5 Mono-Reactive Dye (GE Healthcare), and hybridized to the Human Oligo chip 25k array. Data were collected and normalized using a 3D-Gene Scanner 3000 system (Toray). For global normalization, background value was subtracted, and subsequently adjusted to the average signal value of 25. Each probe on the microarray was linked with specific Gene Ontology (GO) terms based on Oligo MicroArray DataBase system (OMAD) (Operon).

### Promoter assay

The DNA fragment of the *HOXB4* gene promoter region, including 2 transcriptional start sites [20], was obtained from human genomic DNA of a healthy volunteer by PCR using the following primers: forward: 5′-CGCAGGTACCCTATGTAAATCCTGGTGT-3′ (spanning positions −262 to −279 from the translational start site); reverse: 5′-ACCCCTCGAGCGTTTTCCTGTTTCCGAA-3′ (−55 to −72 from the translational start site). These primers contain artificial restriction enzyme sites (*Kpn*I and *Xho*I, respectively), and the amplified DNA fragments were inserted into the *Kpn*I/*Xho*I sites of pGL3-Basic (Promega). GATA deletion construct was generated using a QuickChange™ Site-Directed Mutagenesis Kit (Stratagene) using the mutagenesis primer 5′-GTGAGCAGATTTCCTCGGGAATCGCAGGCG-3′.

To assay *HOXB4* transcriptional activity, aliquots of 5×10^5^ K562 cells (ATCC CCL-243) [19] were transfected with 1 ug of *HOXB4* promoter construct and 50 ng of a *Renilla* luciferase reporter plasmid with FuGene 6 (Promega). After 24 h of incubation in culture media, the cells were harvested, and both firefly and *Renilla* luciferase activities in the cell extracts were determined using the Dual Luciferase Reporter Assay System (Promega).

### Electrophoretic mobility assay (EMSA)

Nuclear extracts were prepared by the method of Andrews and Faller [21]. EMSA analysis was conducted essentially as described previously [22]. The oligonucleotide sequences for the GATA site at −160/−157 were as follows: forward, 5′-GATTTCCTTATCCGGGAATC-3′; reverse, 5′-GATTCCCGGATAAGGAAATC-3′ (wild-type); and forward, 5′-GATTTCCTAAACGGGAATC-3′; reverse, 5′-GATTCCCGTTTAGGAAATC-3′ (GATA mutated).

### Quantitative ChIP analysis

Real-time-PCR-based quantitative chromatin immunoprecipitation (ChIP) analysis was conducted as described [23], [24]. Primer sequences are available upon request.

### Antibodies

Antibodies for GATA-2 (H-116), Myc (9E10), and His probe (G-18) were obtained from Santa Cruz Biotechnology. Alpha-Tubulin (CP06) was obtained from Calbiochem. GATA-2 (ab22849) and rabbit control IgG were obtained from Abcam.

### Western blotting analysis

Whole-cell extracts were prepared by boiling cells for 5 min in SDS sample buffer, and the samples were resolved by SDS-PAGE and transferred onto Immobilon-P membranes (Millipore), as described previously [24]. Detection was performed with ECL plus (GE Healthcare) and BioMAX XAR Film (Kodak).

### Statistics

Statistical significance was assessed by two-sided Student's *t* test. Potential correlations between *HOXB4* and *GATA-2* expression levels were examined by Spearman's rank correlation test. Z-score was calculated to rank the GO terms by the relative changes in gene expression.

## Results

### Expression profiling identified *HOXB4* as a GATA-2 downstream target gene

To identify GATA-2-regulated genes that may contribute to the pathophysiology of AA, we conducted expression profiling using human cord blood-derived CD34-positive cells infected with control and *GATA-2* lentiviral shRNA. Western blotting and quantitative RT-PCR revealed knockdown of GATA-2 protein and mRNA expression (Figures 1A, 1B). We first selected genes that showed 1) changes of more than 1.4-fold, and 2) more than 20 of normalized expression value, which yielded 1126 downregulated and 572 upregulated genes, respectively (Table S1). Quantitative RT-PCR confirmed significant downregulation of representative GATA-2 target genes, such as *KIT* and *GFI1B* (Figure 1B) [25]. Gene Ontology analysis revealed significant enrichment for “signal transduction,” “regulation of transcription, DNA-dependent,” and “Metabolic process” (Figure 1C). In the present study, we focused on the *HOXB4* gene for further analysis (Figure 1B), because HOXB4 has been reported to play an important role in inducing HSC expansion [26], [27]. Furthermore, it has also been reported that restoring HOXB4 protein in HSCs of AA patients rescues growth potential [28].

### GATA-2 regulates *HOXB4* transcription

To determine whether *GATA-2* and *HOXB4* are functionally linked, we transiently transfected *GATA-2* expression vector into K562 cells using an Amaxa Nucleofector kit (Figure 2A) and examined whether GATA-2 altered *HOXB4* expression. This analysis indicated that GATA-2 significantly induced expression of *HOXB4* (Figure 2B). We also examined whether the stable overexpression of GATA-2 affects *HOXB4* expression. The levels of *GATA-2* and *HOXB4* expression in 28 clones were analyzed by quantitative RT-PCR. Western blotting confirmed Myc/His-tagged GATA-2 protein expression in all 28 clones (Figure 2C). After analyzing *GATA-2* and *HOXB4* expression levels in 28 clones, we examined the correlation between their expression levels using the Spearman's rank correlation method. The analysis revealed a significant correlation between *GATA-2* and *HOXB4* expression (Figure 2D, r = 0.46, p<0.01). In addition, siRNA-mediated knockdown of endogenous GATA-2 in K562 cells significantly reduced *HOXB4* expression (Figures 2E, 2F).

To further confirm whether GATA-2 regulates *HOXB4* expression in human cord blood-derived CD34-positive cells, we conducted both overexpression and knockdown of GATA-2, using retroviral vector (MSCV) and siRNA, respectively, and confirmed that GATA-2 regulates HOXB4 expression (Figure 2G and Figure S1).

### GATA-2 binds directly to the *HOXB4* promoter and regulates its activity

Our recent ChIP-seq (chromatin immunoprecipitation and sequencing) analysis revealed a significant GATA-2, but not GATA-1, peak at the *HOXB4* promoter in K562 cells (Figure 3A) [24], suggesting that GATA-2 directly regulates *HOXB4* transcription through its promoter. Thus, we evaluated the sequence of the *HOXB4* promoter and focused on the GATA sequence at −160/−157 (Figure 3B), which is conserved between human and mouse [20], and also across species based on the UCSC genome browser (http://genome.ucsc.edu/). However, the surrounding sequence (GGATAA) does not completely coincide with a “canonical” GATA-1 binding motif, such as (A/T)GATA(A/G) or (C/G)(A/T)GATAA(G/A/C)(G/A/C) [6], [9], [24]. As it has been demonstrated that the incomplete GATA motif, including GATA(A/G), could be bound by GATA-1 [24], we hypothesized that GATA-2 binds to the GATA sequence at −160/−157 of the *HOXB4* promoter and confers a significant promoter activity. To test this possibility, we first conducted luciferase reporter assay in K562 cells using wild-type and GATA-deleted *HOXB4* promoter constructs. As shown in Figure 3C, deletion of the GATA sequence significantly decreased reporter activity as compared to the wild-type promoter activity. Also, we analyzed if GATA-2 depletion could affect promoter activity of HOXB4. K562 cells were infected with control and *GATA-2* lentiviral shRNA, and subsequently puromucin was added in culture media for selection. Western blotting and quantitative RT-PCR revealed knockdown of GATA-2 protein and mRNA expression (Figures 3D). As shown in Figure 3E, GATA-2 knockdown significantly reduced the promoter activity of HOXB4.

Next, to assess whether GATA-2 binds to the GATA sequence of the *HOXB4* promoter, EMSA analysis was conducted with nuclear extracts from K562 cells transiently overexpressing GATA-2. As shown in Figure 4A, a single major band was detected after incubation of nuclear extracts of K562 cells with a FITC-labeled oligonucleotide containing the −160/−157 GATA sequence (lane 2). The intensity of this band increased in proportion to the extent of GATA-2 overexpression (lanes 3–4) and the band intensity decreased considerably when a 100-fold excess of unlabeled oligonucleotide was included in the binding reaction (lane 5). In contrast, a 100-fold excess of a GATA-mutated oligonucleotide (TATC to AAAC) did not affect DNA binding (lane 6). Quantitative ChIP analysis in both K562 and cord blood-derived CD34-positive cells revealed GATA-2 occupancy at the *HOXB4* promoter region (Figure 4B). As a reference, we also examined GATA-2 occupancy at *GATA-2* −1.8 kb and *NECDIN* promoter regions, which are considered positive and negative control sites, respectively (Figure 4B) [24]. These results suggested that GATA-2 directly regulates *HOXB4* expression via binding to the −160/−157 GATA sequence.

### Significant correlation between *GATA-2* and *HOXB4* expression in clinical samples

To examine correlation between *GATA-2* and *HOXB4* expression, CD34-positive cells were isolated from BM mononuclear cells from 10 patients with AA and 13 patients with ITP after obtaining informed consent. We analyzed *HOXB4* and *GATA-2* mRNA levels by quantitative RT-PCR. As shown in Figure 5, we demonstrated that the expression of *HOXB4* was significantly correlated with that of GATA-2 (r = 0.6573, p<0.01).

## Discussion

In addition to immunological destruction of HSCs, in principle, altered gene expression in HSCs in AA may be an important determinant of AA pathology. Previously, we reported the decreased expression of GATA-2 in CD34-positive BM cells [16], and similar results were observed in an independent study [17]. In the present study, we identified the HOXB4 gene as a direct target of GATA-2 in HSCs. HOXB4 is predominantly expressed in HSCs and is particularly important for controlling HSC proliferation [26], [27], [28], [29]. Therefore, we consider that downregulation of HOXB4 in CD34-positive cells appears to be related to one of the pathophysiological aspects of AA, i.e., a reduced HSC number.

We demonstrated that GATA-2 regulates *HOXB4* mRNA level via direct binding to the −160/−157 GATA site of the *HOXB4* promoter (Figures 2,3,4). However, as it appears that GATA-2 has relatively modest effect on *HOXB4* transcription in our analysis, additional factors may also affect HOXB4 expression. It has been reported that the HOXB4 promoter region from −263 to −116 relative to the translational start site is important for the activity in K562 cells [20], and that thrombopoietin [30], NF-Y [31], and USF1/2 [20], [32] regulate *HOXB4* transcription through the promoter region. It is still unknown whether these factors, including GATA-2, regulate *HOXB4* transcription redundantly or cooperatively in HSCs. NF-Y is known to be a constitutive and ubiquitous transcription factor [33], but it may participate in the regulation of some promoters in a lineage-specific manner by interacting with GATA-1 or GATA-2, such as those of globins, Gfi1b, KLF13, and FcgammaRIIA [34], [35], [36], [37], [38]. Thus, GATA-2 may function in a cooperative manner with NF-Y to induce *HOXB4* in HSCs.

Next, we demonstrated significant correlation between the expression of *HOXB4* and *GATA-2* in CD34-positive cells from patients of AA and ITP (Figure 5), suggesting a functional link between GATA-2 and HOXB4 in clinical samples. Furthermore, in the same series of samples, we also found that both *HOXB4* and *GATA-2* expression levels were significantly lower in CD34-positive cells from AA patients as compared to those from ITP patients (Figure S2). However, ITP patients might have a more active stem and progenitor compartment than normal controls, which would cause the differences in *GATA-2* and *HOXB4* expression to be exaggerated. Thus, further analyses are required to compare the expression of *GATA-2* and *HOXB4* in AA with ITP as opposed to CD34+ cells from healthy donors.

The mechanism underlying the decrease in *GATA-2* expression in CD34-positive cells with AA is unclear. To date, several transcription factors and signaling molecules have been reported to induce and/or maintain *GATA-2* expression in HSCs, such as Scl/TAL1 [24], Evi1 [39], Notch1 [40], BMP, and Wnt signaling [41]. Furthermore, it has been reported that GATA-2 could be a target of epigenetic inactivation by DNA methylation [42]. However, the significance of these factors in the pathogenesis of AA has not yet been evaluated. As shown in Figure 5, the significant correlation between *GATA-2* and *HOXB4* expression also suggested that HOXB4 might regulate GATA-2 expression. Among several studies enumerating candidate target genes of HOXB4 [43], [44], [45], [46], [47], Oshima *et al* [46] and Fan *et al* [47] recently demonstrated that HoxB4 functions as a master regulator of hematopoiesis by directly regulating multiple hematopoietic transcription factors, including Runx1, Scl/TAL1 and Gata2, through integrated analysis of HoxB4 binding and gene expression profiling in murine embryonic stem (ES) cells. Therefore, the pathogenesis of AA might be caused by a defect in regulatory network among hematopoietic transcription factors, which would result in the decrease of *GATA-2* expression. Alternatively, it is also possible that some cytokines, which are thought to play an role in immune-mediated suppression of HSCs, participate in the regulation of GATA-2. In support of this, Xu *et al*. recently demonstrated that *GATA-2* expression is reduced by interferon-gamma in an in vitro analysis based on mesenchymal stem cells [48]. Further studies are required to elucidate the basis of GATA-2 downregulation in AA.

In summary, our results suggested that GATA-2 directly regulates *HOXB4* expression in hematopoietic stem cells, which may play an important role in the development and/or progression of AA.

## Supporting Information

Figure S1 **siRNA-mediated knockdown of GATA-2 in CD34+ cells derived from cord blood.** siRNA-mediated *GATA-2* knockdown in cord blood-derived CD34-positive cells. Quantitative RT-PCR analysis was performed to detect *GATA-2* and *HOXB4* expression (mean ± SE, n = 3). mRNA levels were normalized relative to 28S. *p<0.05.
(EPS)

Figure S2 ***HOXB4*** **expression is significantly decreased in CD34-positive cells in patients with AA and ITP.** GATA-2 and HOXB4 mRNA levels were examined by quantitative RT-PCR analysis using CD34-positive cells from patients with AA (n = 10) and ITP (n = 13) (Figure 5). The levels of expression of these genes were normalized relative to GAPDH mRNA expression. Student's unpaired *t* test was used for statistical analyses.
(EPS)

Table S1 **Gene expression profiling by GATA-2 knockdown in cord blood-derived CD34-positive cells.** A total of 1,126 upregulated and 572 downregulated genes after GATA-2 knockdown in cord blood-derived CD34-positive cells were shown. Each value was shown as global normalization value as described in Materials and Methods.
(XLSX)
